# Intersectionality and the invisibility of transgender health in the Philippines

**DOI:** 10.1186/s41256-022-00269-9

**Published:** 2022-09-30

**Authors:** Luis Emmanuel A. Abesamis

**Affiliations:** Department of Sociology and Behavioral Sciences, De La Salle University, 2401 Taft Avenue, Manila, 1004 Metro Manila Philippines

**Keywords:** Intersectionality, Health policy, Critical transgender studies, Transgender health, Filipinos, Philippines

## Abstract

Transgender (trans) Filipinos are disproportionately vulnerable to health problems because of the inaccessibility of essential healthcare services resulting from the invisibility and exclusion of trans health in Philippine health and related social institutions. Because of the institutional prejudice and discrimination against trans Filipinos in Philippine society, an intersectional approach presents an opportunity to analyze the invisibility and potentially elucidate the unique health needs of trans Filipinos. This article elucidates how the invisibility of trans Filipinos in health is a product of co-existing and interacting prejudiced and discriminatory institutions, such as the law, education, and medicine, where the historical experiences of colonization, the hegemony of cisgenderism, and the impact of capitalism remain salient. By elucidating these co-existing and interacting structures and forces, this article highlights the gaps in the Philippine healthcare system, such as the lack of affirming and protective policies for trans health and the limited cultural competence of healthcare providers. In light of these, future research and policy work must work towards integrating gender-specific and gender-inclusive approaches, centering the voices of trans Filipinos in health discourses, and decolonizing and expanding the local understanding of trans health among Filipinos.

## Introduction

Despite the Philippines being one of the more LGBTQI + friendly countries in Asia, prejudice and discrimination based on an individual’s sexual orientation, gender identity and expression, and sex characteristics (SOGIESC) remain systemic, commonplace, and entrenched within Philippine society [1]. SOGIESC-based prejudice and discrimination (SPD) position the LGBTQI + community as second-class citizens. This minority status makes them more vulnerable to health problems and ill-being [2]. However, this vulnerability is not necessarily similarly experienced.

Transgender (trans) Filipinos are disproportionately vulnerable to health problems because of the inaccessibility of essential services to the local trans community. To illustrate, the unique needs of trans Filipinos vis-à-vis HIV/AIDS remain unaddressed because of the generalization of the vulnerability to HIV/AIDS across key populations under the MSM label [3]. Similarly, despite the vulnerability of the LGBTQI + community to mental health problems, LGBTQI + responsive mental health care remains scarce and inaccessible in the Philippines [1]. Likewise, trans Filipinos engage in self-medicating practices, consuming hormones that they purchase from unregulated markets because of the inaccessibility of culturally-competent healthcare providers and the expensive costs of hormones from regulated providers (e.g., pharmacies; hospitals) [4]. Furthermore, the expensive costs and limited providers of gender-affirming surgeries in the Philippines have substantially limited access to these procedures among trans Filipinos [4]. Because of these, trans Filipinos avoid the formal healthcare system and pursue alternative pathways to healthcare [4].

Local non-government organizations (NGOs), such as The LoveYourself Inc., have made substantial progress in providing and improving the access of trans Filipinos all over the country to trans-affirmative healthcare services, such as free HIV testing, referrals to culturally competent specialists (e.g., endocrinologists, counselors), and provision of gender-affirming hormone therapy. The transition to online modes of service provision during the COVID-19 pandemic further expanded their reach to trans communities outside Metro Manila and Cebu [4]. Further, the Philippine government, NGOs, and relevant service providers are currently developing a national transgender health framework that is envisioned to help inform the provision of trans-specific and affirmative healthcare delivery in the country.

While these national and local initiatives for trans health are welcomed, the invisibility and exclusion of trans Filipinos in policies and programs intended to address their health problems must be analyzed vis-à-vis relevant structures of domination or ideological and systemic patterns of power relations to further contextualized our understanding of trans health among Filipinos [5]. An intersectional approach presents an opportunity to analyze the invisibility and potentially elucidate the unique health needs of trans Filipinos, especially since our understanding of trans health has largely been dominated by Western discourses on LGBTQI + health and is focused on HIV/AIDS, mental health, and gender-affirming services. As such, this article aims to elucidate the salient structures and institutes that contribute to and cultivate the oppression of trans Filipinos within local health and medical settings. In the succeeding sections, I map some of the oppressive structures and institutions that influence trans health among Filipinos.

## Colonialism, cisgenderism, and capitalism as structures of domination

The health inequities experienced by trans Filipinos must be contextualized vis-à-vis colonization’s impact on Philippine society. Aside from the pronounced gender egalitarianism, the spiritual leaders in pre-colonial Philippines (babaylans) were either females or men who “transformed” to be more “effeminate” [6]. However, Spanish colonization disrupted these norms and laid the groundwork for the stigmatization of gender-diverse identities in Philippine society. The impact of colonization remains salient because of the continued privileging of heterocissexist norms of gender and sexuality among Filipinos.

The influence of cisgenderism must also be acknowledged. The disruption of biology-based gender ideology by trans individuals results in tensions that make trans individuals more “visible to stigma” [7]. This visibility contributes to their invisibility in health, especially in medicine where the use of sex characteristics remains a significant identifier and mechanism for categorization. For intance, the use of MSM as a category and its inclusion of trans women [3] hints at the invisibilizing impact that cisgenderism has on trans health.

Trans Filipinos face workplace discrimination, being refused employment or promotion unless they present themselves according to their sex assigned at birth [1]. If trans Filipinos are allowed to work, such economic affordances must be evaluated vis-à-vis how neoliberal capitalist systems commodify their capacity for productive economic work, which oftentimes entails other forms of social and emotional labor in the workplace that cisgender co-workers do not have to do [8]. These illustrate how the capitalist system in Philippine society oppresses trans Filipinos not only through the unequal distribution of economic opportunities among all Filipinos but by forcing them to negotiate their trans identity as well.

## Religion, law, and education as institutions of oppression

In contemporary Philippine society, the influence of the identified structures of domination is especially visible in the creation, passage, and implementation of health and public policies on gender and sexuality. The roles of organized religion and the legal system are salient. For instance, the passage and creation of The Responsible Parenthood and Reproductive Health Act of 2012 were heavily influenced by the Catholic Church, ensuring that heteronormative values such as family formation and marriage remained central [9]. Likewise, the Anti-Discrimination Bill, which may help mitigate health-related SPD, remains unpassed after being on Congress’s agenda for over twenty years due to pushback from religious and conservative groups [9]. Although several local anti-discrimination policies have been enacted, these hardly address the systemic prejudice against the LGBTQI + community that trans Filipinos are disproportionately vulnerable to.

Likewise, the invisibility of trans health in health and medical education in the country creates challenges among Filipino healthcare workers in providing quality healthcare for their trans clients [3]. While some healthcare providers have limited or no knowledge of trans-specific healthcare (e.g., gender-affirming hormone therapy), others have also been enablers and perpetrators of stigma and discrimination (e.g., deadnaming trans clients; refusing to provide services). This is further compounded by the representation of trans identities in psychological and psychiatric sciences. For instance, the continued inclusion of gender dysphoria—preceded by gender identity disorder and transsexualism—as a mental disorder in the DSM surfaces concerns about the continued stigmatization and medicalization of trans individuals, even as they access supposedly gender-affirming services (e.g., counseling before transitioning) [10].

## Future directions for trans health research and policy in the Philippines

In elucidating these co-existing ideological structures of domination and their manifestations in Philippine social institutions, the gaps in the Philippine healthcare system vis-à-vis addressing trans-specific health needs and problems are further reified. There is an opportunity for future research and policy work in the Philippines to address these gaps. Future work can begin their efforts by focusing on the following future directions:

- *Gender-inclusive and gender-specific approaches to trans health* Future research and policy work can explore the applications and implications of both gender-inclusive and gender-specific approaches to trans health in the Philippines. Research deploying both methodologies may further elucidate shared experiences across the LGBTQI + community without compromising the unique, intersecting experiences of oppression that inform trans health among Filipinos. Similarly, adopting both approaches may contribute to mainstreaming trans health issues into the greater health discourse while also demanding for greater affordances for trans Filipinos in health.
- *Centering of trans Filipino voices in health discourses* Future efforts must center the experiences of the trans community in the Philippines in public health discourses in both academic and policymaking circles. Although local LGBTQI + organizations are actively involved in the deliberations of both anti-discrimination legislations, the participation of the trans community in the health policy development process as co-creators, implementors, and consultants must be ensured as well. This would facilitate mechanisms for transparency and accountability that greatly empower marginalized sectors. The academe, especially in the health and medical sciences, must also be intentional in amplifying the voices of trans Filipino scholars.
- *Decolonizing and expanding the scope of trans health* Health research and policy work in the Philippines must make efforts to push the envelope and explore trans health beyond its current scope [6]. Filipino scholars must be cognizant of how global, particularly Western, discourses on trans health shape local discourses within both academic and policymaking arenas. Further, local research must consider exploring health experiences beyond sexual and mental health among the trans community. Though important and relevant issues, general health studies would create an impetus for the Philippine healthcare system to pursue improvements in its policies and programs.

## Conclusions

An intersectional approach has shed light on the nature and complexity of trans health inequities among Filipinos. The invisibility of trans Filipinos in health is a product of co-existing and interacting prejudicial and discriminatory institutions, such as the law, education, and medicine, where the history of colonization, the hegemony of cisgenderism, and the impact of capitalism remain salient. In light of this, there is an opportunity for policy and research work on trans health to address the gaps in the Philippine healthcare system. As such, the roles of both academic and policymaking circles are apparent. While much work is yet to be done, these initiatives contribute to the development and cultivation of a more inclusive and responsive healthcare system—one where trans Filipinos are not only represented but also heard.

## Data Availability

Not applicable.

## Abbreviations

LGBTQI+: Lesbian, Gay, Bisexual, Trans, Queer, Intersex,+
SOGIESC: Sexual orientation, gender identity and expression, sex characteristics
SPD: SOGIESC-based Prejudice and Discrimination
HIV: Human Immunodeficiency Virus
AIDS: Acquired Immunodeficiency Syndrome
MSM: Men who have sex with men
NGO: Non-government organizations
DSM: Diagnostic and statistical manual
